# (*Z*)-3-(4-Chloro­phen­yl)-1-(2,4-difluoro­phen­yl)-2-(1*H*-1,2,4-triazol-1-yl)prop-2-en-1-one

**DOI:** 10.1107/S1600536812022118

**Published:** 2012-05-19

**Authors:** Xin-Mei Peng, Ben-Tao Yin, Cheng-He Zhou

**Affiliations:** a Laboratory of Bioorganic & Medicinal Chemistry, School of Chemistry and Chemical Engineering, Southwest University, Chongqing 400715, People’s Republic of China

## Abstract

The asymmetric unit of the title compound, C_17_H_10_ClF_2_N_3_O, contains three independent mol­ecules. In each mol­ecule, the C=C bond has a *cis* conformation with respect to the triazole and chloro­phenyl groups. The dihedral angles formed by the triazole ring with the diflurophenyl and chloro­phenyl benzene rings, respectively, are 20.10 (14) and 73.22 (15), 25.31 (15) and 84.44 (15), and 16.44 (13) and 61.72 (14)° in the three mol­ecules while the dihedral angles between the benzene rings are 66.54 (13), 85.82 (12) and 58.37 (12)°.

## Related literature

For applications of triazole compounds in chemistry and medicinal chemistry, see: Bai *et al.* (2007 ▶); Chang *et al.* (2011 ▶); Wang & Zhou (2011 ▶); Zhou & Wang (2012 ▶). For the pharmacological activity of chalcones, see: Jin *et al.* (2010 ▶). For the synthesis of the title compound, see: Yan *et al.* (2009 ▶). For related structures, see: Wang *et al.* (2009 ▶); Yan *et al.* (2009 ▶).
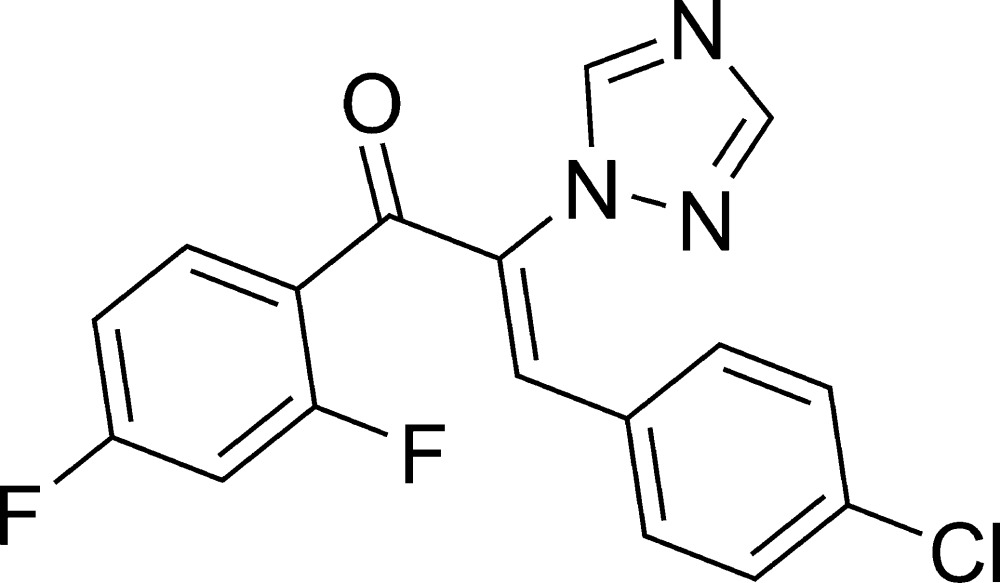


## Experimental

### 

#### Crystal data

C_17_H_10_ClF_2_N_3_O
*M* *_r_* = 345.73
Monoclinic, 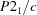

*a* = 17.237 (3) Å
*b* = 10.466 (2) Å
*c* = 26.554 (5) Å
β = 102.378 (4)°
*V* = 4679.2 (16) Å^3^
*Z* = 12
Mo *K*α radiation
μ = 0.28 mm^−1^
*T* = 296 K
0.23 × 0.21 × 0.17 mm

#### Data collection

Bruker APEXII CCD diffractometer
Absorption correction: multi-scan (*SADABS*; Bruker, 2009 ▶) *T*_min_ = 0.939, *T*_max_ = 0.955
25352 measured reflections
9200 independent reflections
5656 reflections with *I* > 2σ(*I*)
*R*_int_ = 0.032

#### Refinement

*R*[*F* ^2^ > 2σ(*F* ^2^)] = 0.045
*wR*(*F* ^2^) = 0.133
*S* = 1.01
9200 reflections
650 parameters
H-atom parameters constrained
Δρ_max_ = 0.34 e Å^−3^
Δρ_min_ = −0.35 e Å^−3^

### 

Data collection: *APEX2* (Bruker, 2009 ▶); cell refinement: *SAINT* (Bruker, 2009 ▶); data reduction: *SAINT*; program(s) used to solve structure: *SHELXS97* (Sheldrick, 2008 ▶); program(s) used to refine structure: *SHELXL97* (Sheldrick, 2008 ▶); molecular graphics: *PLATON* (Spek, 2009 ▶); software used to prepare material for publication: *SHELXTL* (Sheldrick, 2008 ▶).

## Supplementary Material

Crystal structure: contains datablock(s) global, I. DOI: 10.1107/S1600536812022118/lh5474sup1.cif

Structure factors: contains datablock(s) I. DOI: 10.1107/S1600536812022118/lh5474Isup2.hkl

Supplementary material file. DOI: 10.1107/S1600536812022118/lh5474Isup3.cml

Additional supplementary materials:  crystallographic information; 3D view; checkCIF report

## Figures and Tables

**Fig. 1.**
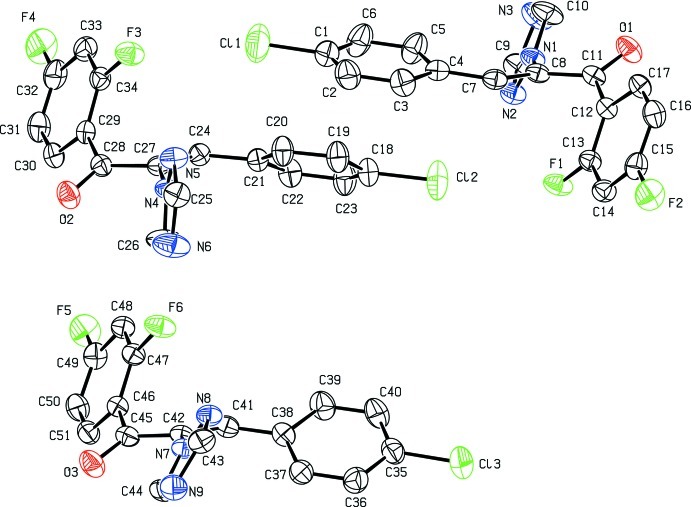
The asymmetric unit of (I), showing the displacement ellipsoids drawn at the 30% probability level.

## supplementary crystallographic information

### Comment

Triazole-based derivatives exhibit extensive potential applications in chemical, medincinal, biological, supramolecular and material sciences (Bai *et al.*, 2007; Chang *et al.*, 2011; Wang *et al.*, 2011; Zhou *et al.*, 2012) and therefore related research has been attracting great attention. Our interest is to invesigate the hybrids of triazole with chalcones (Jin *et al.*, 2010). Some triazole-based chalcones have been reported in our group (Wang *et al.*, 2009; Yan *et al.*, 2009). Herein, the crystal structure of the title compound (I) is reported.

The asymmetric unit of (I) is shown in Fig. 1. In each, the C═C bond has a cis conformation with respect the the triazole and chlorophenyl groups. The dihedral angles formed in each molecule by the triazole ring with the diflurophenyl and chlorophenyl benzene rings respectively, is 20.10 (14) and 73.22 (15) Å [N1/N2/N3/C9/C10 with C12-C17 and C1-C6], 25.31 (15) and 84.44 (15)Å [N4/N5/N6/C25/C26 with C29-C34 and C18-C23], 16.44 (13) and 61.72 (14)Å [N7/N8/N9/C43C44 with C46-C51 and C35-C40]. The dihedral angles between the two benzene rings in each molecule is 66.54 (13) [C1-C6 and C12-C17], 85.82 (12)Å [C18-C23 and C29-C34] and 58.37 (12)Å [C35-C40 and C46-C51].

### Experimental

The title compound was prepared according to the procedure of Yan *et al.* (2009). Single crystals were grown from slow evaporation of a solution of (I) in ethyl acetate and petroleum ether (1:10, V/V) at room temperature.

### Refinement

H atoms were placed in calculated positions with C—H = 0.93 Å. The *U*_iso_(H) value was set equal to 1.2U_eq_(C).

### Crystal data

**Table:**

C_17_H_10_ClF_2_N_3_O	*F*(000) = 2112
*M**_r_* = 345.73	*D*_x_ = 1.472 Mg m^−^^3^
Monoclinic, *P*2_1_/*c*	Mo *K*α radiation, λ = 0.71073 Å
Hall symbol: -P 2ybc	Cell parameters from 5332 reflections
*a* = 17.237 (3) Å	θ = 2.2–22.9°
*b* = 10.466 (2) Å	µ = 0.28 mm^−^^1^
*c* = 26.554 (5) Å	*T* = 296 K
β = 102.378 (4)°	Block, colorless
*V* = 4679.2 (16) Å^3^	0.23 × 0.21 × 0.17 mm
*Z* = 12	

### Data collection

**Table:**

Bruker APEXII CCD diffractometer	9200 independent reflections
Radiation source: fine-focus sealed tube	5656 reflections with *I* > 2σ(*I*)
Graphite monochromator	*R*_int_ = 0.032
φ and ω scans	θ_max_ = 26.0°, θ_min_ = 1.6°
Absorption correction: multi-scan (*SADABS*; Bruker, 2009)	*h* = −20→21
*T*_min_ = 0.939, *T*_max_ = 0.955	*k* = −12→10
25352 measured reflections	*l* = −32→30

### Refinement

**Table:**

Refinement on *F*^2^	Secondary atom site location: difference Fourier map
Least-squares matrix: full	Hydrogen site location: inferred from neighbouring sites
*R*[*F*^2^ > 2σ(*F*^2^)] = 0.045	H-atom parameters constrained
*wR*(*F*^2^) = 0.133	*w* = 1/[σ^2^(*F*_o_^2^) + (0.0626*P*)^2^ + 0.7017*P*] where *P* = (*F*_o_^2^ + 2*F*_c_^2^)/3
*S* = 1.01	(Δ/σ)_max_ = 0.001
9200 reflections	Δρ_max_ = 0.34 e Å^−^^3^
650 parameters	Δρ_min_ = −0.35 e Å^−^^3^
0 restraints	Extinction correction: *SHELXL97* (Sheldrick, 2008), Fc^*^=kFc[1+0.001xFc^2^λ^3^/sin(2θ)]^-1/4^
Primary atom site location: structure-invariant direct methods	Extinction coefficient: 0.0015 (2)

### Special details

**Table:**

Geometry. All e.s.d.'s (except the e.s.d. in the dihedral angle between two l.s. planes) are estimated using the full covariance matrix. The cell e.s.d.'s are taken into account individually in the estimation of e.s.d.'s in distances, angles and torsion angles; correlations between e.s.d.'s in cell parameters are only used when they are defined by crystal symmetry. An approximate (isotropic) treatment of cell e.s.d.'s is used for estimating e.s.d.'s involving l.s. planes.
Refinement. Refinement of *F*^2^ against ALL reflections. The weighted *R*-factor *wR* and goodness of fit *S* are based on *F*^2^, conventional *R*-factors *R* are based on *F*, with *F* set to zero for negative *F*^2^. The threshold expression of *F*^2^ > σ(*F*^2^) is used only for calculating *R*-factors(gt) *etc*. and is not relevant to the choice of reflections for refinement. *R*-factors based on *F*^2^ are statistically about twice as large as those based on *F*, and *R*- factors based on ALL data will be even larger.

### Fractional atomic coordinates and isotropic or equivalent isotropic displacement parameters (Å^2^)

**Table:**

	*x*	*y*	*z*	*U*_iso_*/*U*_eq_	
F1	0.20468 (8)	0.35283 (12)	0.14981 (5)	0.0668 (4)	
F2	0.05967 (9)	0.66430 (13)	0.21328 (7)	0.0874 (5)	
F3	0.78892 (9)	0.56839 (13)	0.52214 (5)	0.0708 (4)	
F4	0.92930 (10)	0.25782 (15)	0.45456 (8)	0.1080 (6)	
F5	0.92861 (9)	0.25932 (13)	0.09951 (7)	0.0901 (5)	
F6	0.80518 (9)	0.56279 (12)	0.18279 (5)	0.0717 (4)	
C1	0.52331 (16)	0.0907 (3)	0.29462 (11)	0.0747 (7)	
C2	0.49247 (17)	0.2060 (3)	0.30181 (12)	0.0815 (8)	
H2A	0.5243	0.2694	0.3202	0.098*	
C3	0.41327 (15)	0.2288 (2)	0.28162 (10)	0.0694 (7)	
H3A	0.3922	0.3083	0.2868	0.083*	
C4	0.36428 (13)	0.1379 (2)	0.25404 (8)	0.0509 (5)	
C5	0.39820 (17)	0.0207 (2)	0.24830 (11)	0.0806 (8)	
H5A	0.3668	−0.0441	0.2306	0.097*	
C6	0.47705 (18)	−0.0022 (3)	0.26816 (13)	0.0909 (9)	
H6A	0.4988	−0.0815	0.2635	0.109*	
C7	0.28127 (13)	0.1712 (2)	0.23551 (8)	0.0506 (5)	
H7A	0.2678	0.2520	0.2454	0.061*	
C8	0.22060 (13)	0.10637 (19)	0.20677 (8)	0.0474 (5)	
C9	0.26059 (15)	−0.1494 (2)	0.13538 (10)	0.0628 (7)	
H9A	0.2812	−0.1870	0.1093	0.075*	
C10	0.20371 (17)	−0.1289 (2)	0.19568 (12)	0.0769 (8)	
H10A	0.1755	−0.1442	0.2213	0.092*	
C11	0.13747 (13)	0.1504 (2)	0.19983 (8)	0.0492 (5)	
C12	0.11973 (12)	0.2886 (2)	0.20359 (8)	0.0460 (5)	
C13	0.15149 (13)	0.3843 (2)	0.17871 (8)	0.0480 (5)	
C14	0.13213 (14)	0.5102 (2)	0.18068 (9)	0.0560 (6)	
H14A	0.1541	0.5723	0.1629	0.067*	
C15	0.07866 (14)	0.5404 (2)	0.21011 (10)	0.0604 (6)	
C16	0.04492 (14)	0.4512 (2)	0.23620 (10)	0.0637 (7)	
H16A	0.0093	0.4750	0.2563	0.076*	
C17	0.06485 (13)	0.3261 (2)	0.23190 (9)	0.0566 (6)	
H17A	0.0408	0.2641	0.2485	0.068*	
C18	0.45770 (14)	0.8091 (3)	0.38261 (11)	0.0663 (7)	
C19	0.49635 (14)	0.8600 (2)	0.42844 (11)	0.0712 (7)	
H19A	0.4688	0.9098	0.4478	0.085*	
C20	0.57613 (14)	0.8375 (2)	0.44587 (10)	0.0660 (7)	
H20A	0.6026	0.8735	0.4768	0.079*	
C21	0.61753 (12)	0.76197 (19)	0.41791 (9)	0.0496 (5)	
C22	0.57591 (14)	0.7066 (2)	0.37302 (10)	0.0659 (7)	
H22A	0.6019	0.6516	0.3547	0.079*	
C23	0.49680 (15)	0.7317 (3)	0.35512 (11)	0.0761 (8)	
H23A	0.4698	0.6959	0.3242	0.091*	
C24	0.70225 (12)	0.7339 (2)	0.43406 (8)	0.0491 (5)	
H24A	0.7171	0.6526	0.4256	0.059*	
C25	0.72815 (15)	1.0924 (2)	0.51513 (10)	0.0626 (6)	
H25A	0.7157	1.1425	0.5412	0.075*	
C26	0.75847 (19)	1.0413 (2)	0.44775 (11)	0.0860 (9)	
H26A	0.7728	1.0434	0.4159	0.103*	
C27	0.76129 (12)	0.80666 (19)	0.45894 (8)	0.0454 (5)	
C28	0.84596 (13)	0.7695 (2)	0.46558 (8)	0.0495 (5)	
C29	0.86721 (12)	0.6328 (2)	0.46420 (8)	0.0478 (5)	
C30	0.92034 (13)	0.5955 (2)	0.43433 (10)	0.0598 (6)	
H30A	0.9422	0.6573	0.4164	0.072*	
C31	0.94140 (15)	0.4701 (3)	0.43059 (11)	0.0717 (7)	
H31A	0.9762	0.4463	0.4099	0.086*	
C32	0.91002 (15)	0.3813 (2)	0.45796 (11)	0.0697 (7)	
C33	0.85911 (14)	0.4115 (2)	0.48906 (10)	0.0621 (6)	
H33A	0.8393	0.3494	0.5080	0.075*	
C34	0.83867 (12)	0.5369 (2)	0.49113 (9)	0.0507 (5)	
C35	0.48083 (14)	0.8658 (3)	0.05895 (10)	0.0672 (7)	
C36	0.53758 (16)	0.9580 (3)	0.06771 (10)	0.0712 (7)	
H36A	0.5232	1.0434	0.0623	0.085*	
C37	0.61618 (15)	0.9259 (2)	0.08456 (10)	0.0654 (7)	
H37A	0.6545	0.9899	0.0905	0.078*	
C38	0.63907 (13)	0.8001 (2)	0.09278 (9)	0.0535 (6)	
C39	0.58004 (16)	0.7084 (3)	0.08339 (11)	0.0749 (8)	
H39A	0.5940	0.6227	0.0882	0.090*	
C40	0.50156 (16)	0.7403 (3)	0.06715 (12)	0.0795 (8)	
H40A	0.4627	0.6771	0.0618	0.095*	
C41	0.72159 (13)	0.7588 (2)	0.10696 (9)	0.0537 (6)	
H41A	0.7306	0.6763	0.0965	0.064*	
C42	0.78625 (13)	0.81873 (19)	0.13225 (8)	0.0486 (5)	
C43	0.74745 (15)	1.0733 (2)	0.20480 (10)	0.0635 (7)	
H43A	0.7235	1.1114	0.2292	0.076*	
C44	0.81640 (15)	1.0507 (2)	0.15076 (10)	0.0627 (7)	
H44A	0.8507	1.0644	0.1286	0.075*	
C45	0.86757 (14)	0.7726 (2)	0.13301 (9)	0.0529 (6)	
C46	0.88146 (12)	0.6337 (2)	0.12470 (8)	0.0470 (5)	
C47	0.85120 (13)	0.5356 (2)	0.14879 (8)	0.0507 (5)	
C48	0.86624 (13)	0.4091 (2)	0.14169 (9)	0.0555 (6)	
H48A	0.8453	0.3444	0.1588	0.067*	
C49	0.91392 (14)	0.3839 (2)	0.10796 (10)	0.0605 (6)	
C50	0.94698 (14)	0.4753 (3)	0.08309 (10)	0.0646 (7)	
H50A	0.9795	0.4536	0.0606	0.078*	
C51	0.93108 (13)	0.6008 (2)	0.09209 (9)	0.0579 (6)	
H51A	0.9541	0.6650	0.0760	0.069*	
Cl1	0.62306 (5)	0.05800 (11)	0.31895 (4)	0.1276 (4)	
Cl2	0.35746 (4)	0.84070 (10)	0.36066 (4)	0.1091 (3)	
Cl3	0.38200 (4)	0.90807 (9)	0.03691 (4)	0.0978 (3)	
N1	0.22974 (11)	−0.01526 (15)	0.18508 (7)	0.0500 (5)	
N2	0.26661 (11)	−0.02714 (17)	0.14488 (7)	0.0547 (5)	
N3	0.22279 (14)	−0.21717 (19)	0.16551 (10)	0.0817 (7)	
N4	0.74910 (10)	0.93572 (15)	0.47265 (7)	0.0467 (4)	
N5	0.72995 (12)	0.96812 (17)	0.51768 (7)	0.0587 (5)	
N6	0.74517 (17)	1.1429 (2)	0.47318 (11)	0.0942 (8)	
N7	0.78198 (11)	0.93912 (15)	0.15638 (7)	0.0489 (4)	
N8	0.73630 (11)	0.95284 (17)	0.19205 (7)	0.0548 (5)	
N9	0.79562 (13)	1.13848 (18)	0.18044 (9)	0.0696 (6)	
O1	0.08400 (10)	0.07287 (15)	0.19375 (7)	0.0703 (5)	
O2	0.89650 (9)	0.85179 (15)	0.46908 (7)	0.0690 (5)	
O3	0.92184 (10)	0.84789 (16)	0.13720 (7)	0.0728 (5)	

### Atomic displacement parameters (Å^2^)

**Table:**

	*U*^11^	*U*^22^	*U*^33^	*U*^12^	*U*^13^	*U*^23^
F1	0.0852 (10)	0.0554 (8)	0.0731 (9)	0.0047 (7)	0.0466 (8)	0.0060 (7)
F2	0.0891 (11)	0.0574 (9)	0.1196 (14)	0.0236 (8)	0.0306 (10)	−0.0069 (8)
F3	0.0855 (10)	0.0639 (9)	0.0737 (9)	0.0103 (7)	0.0411 (8)	0.0058 (7)
F4	0.1029 (13)	0.0660 (10)	0.1600 (18)	0.0361 (9)	0.0389 (12)	−0.0070 (10)
F5	0.0972 (12)	0.0578 (9)	0.1217 (14)	0.0124 (8)	0.0375 (10)	−0.0182 (9)
F6	0.0993 (11)	0.0597 (8)	0.0698 (9)	0.0010 (7)	0.0486 (8)	0.0020 (7)
C1	0.0620 (17)	0.089 (2)	0.0703 (18)	−0.0014 (16)	0.0085 (14)	0.0089 (16)
C2	0.0697 (19)	0.079 (2)	0.093 (2)	−0.0155 (16)	0.0105 (16)	−0.0116 (16)
C3	0.0668 (17)	0.0624 (16)	0.0796 (19)	−0.0030 (13)	0.0170 (14)	−0.0114 (13)
C4	0.0609 (14)	0.0462 (13)	0.0474 (13)	−0.0010 (11)	0.0155 (11)	0.0011 (10)
C5	0.0762 (19)	0.0583 (16)	0.095 (2)	0.0095 (14)	−0.0097 (16)	−0.0104 (15)
C6	0.080 (2)	0.0715 (19)	0.109 (2)	0.0242 (16)	−0.0054 (18)	−0.0060 (17)
C7	0.0635 (15)	0.0403 (12)	0.0516 (13)	0.0008 (11)	0.0203 (11)	0.0007 (10)
C8	0.0603 (14)	0.0358 (11)	0.0504 (13)	−0.0037 (10)	0.0213 (11)	0.0001 (9)
C9	0.0751 (17)	0.0405 (13)	0.0768 (17)	0.0004 (12)	0.0248 (14)	−0.0078 (12)
C10	0.098 (2)	0.0397 (14)	0.108 (2)	−0.0073 (13)	0.0561 (18)	0.0052 (14)
C11	0.0546 (13)	0.0476 (13)	0.0498 (13)	−0.0083 (11)	0.0211 (11)	−0.0019 (10)
C12	0.0453 (12)	0.0454 (12)	0.0489 (13)	−0.0030 (10)	0.0136 (10)	−0.0028 (10)
C13	0.0509 (13)	0.0481 (13)	0.0488 (13)	0.0009 (10)	0.0191 (11)	−0.0019 (10)
C14	0.0609 (15)	0.0461 (13)	0.0628 (15)	0.0013 (11)	0.0169 (12)	0.0022 (11)
C15	0.0559 (15)	0.0523 (15)	0.0716 (16)	0.0128 (12)	0.0104 (13)	−0.0089 (12)
C16	0.0534 (14)	0.0699 (17)	0.0732 (17)	0.0086 (12)	0.0251 (13)	−0.0118 (13)
C17	0.0502 (13)	0.0612 (15)	0.0630 (15)	−0.0028 (11)	0.0221 (12)	−0.0025 (11)
C18	0.0450 (13)	0.0761 (17)	0.0757 (18)	−0.0005 (12)	0.0081 (13)	−0.0074 (14)
C19	0.0517 (15)	0.0742 (17)	0.088 (2)	0.0079 (13)	0.0147 (14)	−0.0202 (15)
C20	0.0500 (14)	0.0764 (17)	0.0701 (17)	0.0039 (13)	0.0099 (12)	−0.0214 (13)
C21	0.0471 (13)	0.0438 (12)	0.0575 (14)	0.0006 (10)	0.0107 (11)	−0.0043 (10)
C22	0.0574 (15)	0.0715 (16)	0.0687 (17)	−0.0019 (13)	0.0131 (13)	−0.0196 (13)
C23	0.0577 (16)	0.096 (2)	0.0688 (18)	−0.0033 (15)	0.0013 (13)	−0.0230 (15)
C24	0.0492 (13)	0.0443 (12)	0.0545 (14)	0.0041 (10)	0.0131 (11)	−0.0022 (10)
C25	0.0736 (17)	0.0462 (15)	0.0695 (17)	0.0032 (12)	0.0188 (14)	−0.0100 (12)
C26	0.139 (3)	0.0516 (16)	0.085 (2)	0.0185 (16)	0.062 (2)	0.0197 (15)
C27	0.0529 (13)	0.0390 (12)	0.0460 (12)	0.0029 (10)	0.0141 (10)	0.0009 (9)
C28	0.0470 (13)	0.0525 (13)	0.0493 (13)	−0.0041 (11)	0.0110 (10)	−0.0030 (10)
C29	0.0383 (11)	0.0519 (13)	0.0518 (13)	0.0040 (10)	0.0065 (10)	−0.0028 (10)
C30	0.0436 (13)	0.0695 (16)	0.0689 (16)	0.0039 (12)	0.0178 (12)	0.0007 (12)
C31	0.0531 (15)	0.0789 (19)	0.086 (2)	0.0196 (14)	0.0214 (14)	−0.0113 (16)
C32	0.0599 (16)	0.0566 (16)	0.090 (2)	0.0202 (13)	0.0109 (15)	−0.0109 (14)
C33	0.0591 (15)	0.0538 (15)	0.0720 (17)	0.0072 (12)	0.0107 (13)	0.0035 (12)
C34	0.0461 (13)	0.0543 (14)	0.0530 (13)	0.0073 (10)	0.0133 (11)	−0.0021 (11)
C35	0.0549 (15)	0.0831 (19)	0.0667 (17)	−0.0069 (14)	0.0197 (13)	−0.0031 (14)
C36	0.0665 (17)	0.0618 (16)	0.0816 (19)	0.0002 (14)	0.0075 (14)	0.0038 (14)
C37	0.0626 (16)	0.0576 (15)	0.0721 (17)	−0.0108 (12)	0.0059 (13)	0.0026 (12)
C38	0.0561 (14)	0.0512 (14)	0.0550 (14)	−0.0074 (11)	0.0160 (11)	−0.0104 (11)
C39	0.0683 (18)	0.0578 (15)	0.097 (2)	−0.0115 (13)	0.0143 (15)	−0.0122 (14)
C40	0.0622 (18)	0.077 (2)	0.100 (2)	−0.0216 (15)	0.0173 (16)	−0.0139 (16)
C41	0.0628 (15)	0.0460 (13)	0.0543 (14)	−0.0061 (11)	0.0173 (12)	−0.0040 (10)
C42	0.0592 (14)	0.0384 (12)	0.0502 (13)	−0.0063 (10)	0.0165 (11)	0.0016 (10)
C43	0.0783 (18)	0.0427 (14)	0.0684 (16)	0.0042 (12)	0.0133 (14)	−0.0033 (12)
C44	0.0692 (16)	0.0396 (13)	0.0810 (18)	−0.0086 (12)	0.0201 (14)	0.0077 (12)
C45	0.0592 (15)	0.0503 (13)	0.0510 (14)	−0.0097 (11)	0.0159 (11)	0.0015 (10)
C46	0.0475 (12)	0.0463 (12)	0.0478 (12)	−0.0021 (10)	0.0117 (10)	0.0003 (10)
C47	0.0555 (14)	0.0523 (14)	0.0467 (13)	−0.0020 (11)	0.0164 (11)	−0.0036 (10)
C48	0.0587 (14)	0.0469 (13)	0.0605 (15)	−0.0021 (11)	0.0123 (12)	0.0019 (11)
C49	0.0589 (15)	0.0497 (14)	0.0719 (17)	0.0040 (12)	0.0117 (13)	−0.0118 (12)
C50	0.0548 (15)	0.0729 (18)	0.0718 (17)	0.0045 (13)	0.0260 (13)	−0.0102 (14)
C51	0.0512 (14)	0.0644 (16)	0.0603 (15)	−0.0047 (12)	0.0167 (12)	0.0004 (12)
Cl1	0.0659 (5)	0.1549 (9)	0.1484 (9)	0.0093 (5)	−0.0074 (5)	0.0063 (7)
Cl2	0.0478 (4)	0.1542 (8)	0.1166 (7)	0.0082 (5)	−0.0016 (4)	−0.0248 (6)
Cl3	0.0559 (4)	0.1225 (7)	0.1150 (7)	−0.0003 (4)	0.0183 (4)	0.0045 (5)
N1	0.0602 (12)	0.0345 (10)	0.0607 (12)	−0.0031 (8)	0.0250 (10)	0.0001 (8)
N2	0.0673 (13)	0.0431 (11)	0.0587 (12)	−0.0010 (9)	0.0246 (10)	−0.0015 (9)
N3	0.0952 (17)	0.0379 (11)	0.126 (2)	−0.0095 (11)	0.0542 (16)	−0.0073 (12)
N4	0.0548 (11)	0.0395 (10)	0.0483 (11)	0.0019 (8)	0.0165 (9)	0.0025 (8)
N5	0.0820 (14)	0.0457 (12)	0.0506 (12)	0.0018 (10)	0.0192 (10)	−0.0016 (9)
N6	0.143 (2)	0.0438 (13)	0.114 (2)	0.0119 (13)	0.0683 (19)	0.0111 (13)
N7	0.0567 (11)	0.0364 (10)	0.0544 (11)	−0.0046 (8)	0.0138 (9)	0.0038 (8)
N8	0.0693 (13)	0.0418 (11)	0.0559 (12)	0.0011 (9)	0.0193 (10)	0.0016 (9)
N9	0.0769 (15)	0.0384 (11)	0.0924 (16)	−0.0040 (10)	0.0157 (13)	0.0009 (11)
O1	0.0655 (11)	0.0531 (10)	0.0987 (14)	−0.0161 (9)	0.0317 (10)	−0.0087 (9)
O2	0.0545 (10)	0.0589 (10)	0.0935 (13)	−0.0102 (8)	0.0158 (9)	−0.0070 (9)
O3	0.0633 (11)	0.0564 (10)	0.1024 (14)	−0.0139 (9)	0.0257 (10)	−0.0045 (9)

### Geometric parameters (Å, º)

**Table:**

F1—C13	1.356 (2)	C25—N6	1.321 (3)
F2—C15	1.345 (3)	C25—H25A	0.9300
F3—C34	1.351 (2)	C26—N6	1.306 (3)
F4—C32	1.343 (3)	C26—N4	1.315 (3)
F5—C49	1.356 (3)	C26—H26A	0.9300
F6—C47	1.354 (2)	C27—N4	1.426 (3)
C1—C6	1.354 (4)	C27—C28	1.484 (3)
C1—C2	1.349 (4)	C28—O2	1.214 (2)
C1—Cl1	1.736 (3)	C28—C29	1.479 (3)
C2—C3	1.376 (4)	C29—C30	1.390 (3)
C2—H2A	0.9300	C29—C34	1.383 (3)
C3—C4	1.374 (3)	C30—C31	1.371 (3)
C3—H3A	0.9300	C30—H30A	0.9300
C4—C5	1.382 (3)	C31—C32	1.361 (4)
C4—C7	1.452 (3)	C31—H31A	0.9300
C5—C6	1.369 (4)	C32—C33	1.365 (3)
C5—H5A	0.9300	C33—C34	1.363 (3)
C6—H6A	0.9300	C33—H33A	0.9300
C7—C8	1.339 (3)	C35—C36	1.358 (3)
C7—H7A	0.9300	C35—C40	1.367 (4)
C8—N1	1.420 (3)	C35—Cl3	1.736 (3)
C8—C11	1.479 (3)	C36—C37	1.374 (3)
C9—N2	1.304 (3)	C36—H36A	0.9300
C9—N3	1.339 (3)	C37—C38	1.378 (3)
C9—H9A	0.9300	C37—H37A	0.9300
C10—N3	1.310 (3)	C38—C39	1.382 (3)
C10—N1	1.323 (3)	C38—C41	1.457 (3)
C10—H10A	0.9300	C39—C40	1.370 (4)
C11—O1	1.212 (2)	C39—H39A	0.9300
C11—C12	1.486 (3)	C40—H40A	0.9300
C12—C13	1.376 (3)	C41—C42	1.329 (3)
C12—C17	1.385 (3)	C41—H41A	0.9300
C13—C14	1.363 (3)	C42—N7	1.423 (3)
C14—C15	1.367 (3)	C42—C45	1.479 (3)
C14—H14A	0.9300	C43—N8	1.308 (3)
C15—C16	1.364 (3)	C43—N9	1.343 (3)
C16—C17	1.365 (3)	C43—H43A	0.9300
C16—H16A	0.9300	C44—N9	1.310 (3)
C17—H17A	0.9300	C44—N7	1.332 (3)
C18—C19	1.365 (3)	C44—H44A	0.9300
C18—C23	1.362 (3)	C45—O3	1.210 (3)
C18—Cl2	1.733 (2)	C45—C46	1.497 (3)
C19—C20	1.374 (3)	C46—C47	1.371 (3)
C19—H19A	0.9300	C46—C51	1.385 (3)
C20—C21	1.383 (3)	C47—C48	1.370 (3)
C20—H20A	0.9300	C48—C49	1.365 (3)
C21—C22	1.380 (3)	C48—H48A	0.9300
C21—C24	1.461 (3)	C49—C50	1.356 (3)
C22—C23	1.370 (3)	C50—C51	1.373 (3)
C22—H22A	0.9300	C50—H50A	0.9300
C23—H23A	0.9300	C51—H51A	0.9300
C24—C27	1.328 (3)	N1—N2	1.360 (2)
C24—H24A	0.9300	N4—N5	1.350 (2)
C25—N5	1.303 (3)	N7—N8	1.363 (2)
			
C6—C1—C2	120.6 (3)	C30—C29—C34	116.2 (2)
C6—C1—Cl1	118.5 (2)	C30—C29—C28	118.9 (2)
C2—C1—Cl1	120.8 (2)	C34—C29—C28	124.89 (19)
C3—C2—C1	119.2 (3)	C29—C30—C31	121.8 (2)
C3—C2—H2A	120.4	C29—C30—H30A	119.1
C1—C2—H2A	120.4	C31—C30—H30A	119.1
C2—C3—C4	122.2 (3)	C32—C31—C30	118.2 (2)
C2—C3—H3A	118.9	C32—C31—H31A	120.9
C4—C3—H3A	118.9	C30—C31—H31A	120.9
C5—C4—C3	116.6 (2)	C31—C32—F4	119.3 (2)
C5—C4—C7	125.8 (2)	C31—C32—C33	123.1 (2)
C3—C4—C7	117.6 (2)	F4—C32—C33	117.6 (3)
C4—C5—C6	121.4 (3)	C32—C33—C34	116.9 (2)
C4—C5—H5A	119.3	C32—C33—H33A	121.6
C6—C5—H5A	119.3	C34—C33—H33A	121.6
C1—C6—C5	120.0 (3)	F3—C34—C33	117.6 (2)
C1—C6—H6A	120.0	F3—C34—C29	118.71 (19)
C5—C6—H6A	120.0	C33—C34—C29	123.7 (2)
C8—C7—C4	131.6 (2)	C36—C35—C40	120.1 (3)
C8—C7—H7A	114.2	C36—C35—Cl3	119.7 (2)
C4—C7—H7A	114.2	C40—C35—Cl3	120.2 (2)
C7—C8—N1	123.0 (2)	C35—C36—C37	120.3 (2)
C7—C8—C11	122.05 (19)	C35—C36—H36A	119.8
N1—C8—C11	114.69 (19)	C37—C36—H36A	119.8
N2—C9—N3	115.7 (2)	C36—C37—C38	120.9 (2)
N2—C9—H9A	122.2	C36—C37—H37A	119.5
N3—C9—H9A	122.2	C38—C37—H37A	119.5
N3—C10—N1	111.3 (2)	C39—C38—C37	117.4 (2)
N3—C10—H10A	124.4	C39—C38—C41	118.7 (2)
N1—C10—H10A	124.4	C37—C38—C41	123.7 (2)
O1—C11—C8	119.8 (2)	C40—C39—C38	121.8 (3)
O1—C11—C12	119.9 (2)	C40—C39—H39A	119.1
C8—C11—C12	120.18 (18)	C38—C39—H39A	119.1
C13—C12—C17	116.3 (2)	C35—C40—C39	119.4 (2)
C13—C12—C11	124.56 (18)	C35—C40—H40A	120.3
C17—C12—C11	119.10 (19)	C39—C40—H40A	120.3
F1—C13—C14	117.20 (19)	C42—C41—C38	131.1 (2)
F1—C13—C12	118.81 (18)	C42—C41—H41A	114.4
C14—C13—C12	124.0 (2)	C38—C41—H41A	114.4
C15—C14—C13	116.5 (2)	C41—C42—N7	121.8 (2)
C15—C14—H14A	121.8	C41—C42—C45	122.9 (2)
C13—C14—H14A	121.8	N7—C42—C45	115.06 (19)
F2—C15—C14	117.5 (2)	N8—C43—N9	115.9 (2)
F2—C15—C16	119.5 (2)	N8—C43—H43A	122.1
C14—C15—C16	123.0 (2)	N9—C43—H43A	122.1
C15—C16—C17	118.1 (2)	N9—C44—N7	111.2 (2)
C15—C16—H16A	120.9	N9—C44—H44A	124.4
C17—C16—H16A	120.9	N7—C44—H44A	124.4
C16—C17—C12	122.1 (2)	O3—C45—C42	120.0 (2)
C16—C17—H17A	119.0	O3—C45—C46	120.1 (2)
C12—C17—H17A	119.0	C42—C45—C46	119.76 (19)
C19—C18—C23	120.5 (2)	C47—C46—C51	117.0 (2)
C19—C18—Cl2	119.1 (2)	C47—C46—C45	124.74 (19)
C23—C18—Cl2	120.4 (2)	C51—C46—C45	118.3 (2)
C20—C19—C18	119.7 (2)	F6—C47—C46	119.30 (19)
C20—C19—H19A	120.1	F6—C47—C48	116.8 (2)
C18—C19—H19A	120.1	C46—C47—C48	123.9 (2)
C19—C20—C21	120.8 (2)	C47—C48—C49	115.8 (2)
C19—C20—H20A	119.6	C47—C48—H48A	122.1
C21—C20—H20A	119.6	C49—C48—H48A	122.1
C20—C21—C22	118.1 (2)	F5—C49—C50	119.0 (2)
C20—C21—C24	123.7 (2)	F5—C49—C48	117.0 (2)
C22—C21—C24	118.1 (2)	C50—C49—C48	124.0 (2)
C23—C22—C21	120.9 (2)	C49—C50—C51	118.0 (2)
C23—C22—H22A	119.5	C49—C50—H50A	121.0
C21—C22—H22A	119.5	C51—C50—H50A	121.0
C22—C23—C18	119.9 (2)	C46—C51—C50	121.3 (2)
C22—C23—H23A	120.1	C46—C51—H51A	119.3
C18—C23—H23A	120.1	C50—C51—H51A	119.3
C27—C24—C21	129.9 (2)	C10—N1—N2	108.76 (19)
C27—C24—H24A	115.0	C10—N1—C8	130.4 (2)
C21—C24—H24A	115.0	N2—N1—C8	120.77 (16)
N5—C25—N6	115.8 (2)	C9—N2—N1	102.15 (18)
N5—C25—H25A	122.1	C10—N3—C9	102.13 (19)
N6—C25—H25A	122.1	C26—N4—N5	108.30 (18)
N6—C26—N4	111.7 (2)	C26—N4—C27	128.79 (19)
N6—C26—H26A	124.1	N5—N4—C27	122.78 (17)
N4—C26—H26A	124.1	C25—N5—N4	102.26 (19)
C24—C27—N4	122.06 (19)	C26—N6—C25	101.9 (2)
C24—C27—C28	122.56 (19)	C44—N7—N8	108.84 (19)
N4—C27—C28	114.39 (18)	C44—N7—C42	131.2 (2)
O2—C28—C29	120.7 (2)	N8—N7—C42	119.98 (16)
O2—C28—C27	119.6 (2)	C43—N8—N7	101.92 (19)
C29—C28—C27	119.55 (19)	C44—N9—C43	102.13 (19)
			
C6—C1—C2—C3	−0.4 (5)	C28—C29—C34—C33	179.8 (2)
Cl1—C1—C2—C3	179.5 (2)	C40—C35—C36—C37	0.4 (4)
C1—C2—C3—C4	−0.3 (4)	Cl3—C35—C36—C37	−179.2 (2)
C2—C3—C4—C5	1.2 (4)	C35—C36—C37—C38	0.2 (4)
C2—C3—C4—C7	178.6 (2)	C36—C37—C38—C39	0.0 (4)
C3—C4—C5—C6	−1.4 (4)	C36—C37—C38—C41	175.4 (2)
C7—C4—C5—C6	−178.6 (3)	C37—C38—C39—C40	−0.8 (4)
C2—C1—C6—C5	0.2 (5)	C41—C38—C39—C40	−176.3 (2)
Cl1—C1—C6—C5	−179.7 (2)	C36—C35—C40—C39	−1.1 (4)
C4—C5—C6—C1	0.8 (5)	Cl3—C35—C40—C39	178.5 (2)
C5—C4—C7—C8	−5.3 (4)	C38—C39—C40—C35	1.3 (4)
C3—C4—C7—C8	177.5 (2)	C39—C38—C41—C42	−157.0 (3)
C4—C7—C8—N1	−4.1 (4)	C37—C38—C41—C42	27.8 (4)
C4—C7—C8—C11	169.3 (2)	C38—C41—C42—N7	7.8 (4)
C7—C8—C11—O1	−148.9 (2)	C38—C41—C42—C45	−165.9 (2)
N1—C8—C11—O1	25.1 (3)	C41—C42—C45—O3	150.1 (2)
C7—C8—C11—C12	27.3 (3)	N7—C42—C45—O3	−24.0 (3)
N1—C8—C11—C12	−158.77 (18)	C41—C42—C45—C46	−25.4 (3)
O1—C11—C12—C13	−135.5 (2)	N7—C42—C45—C46	160.52 (18)
C8—C11—C12—C13	48.3 (3)	O3—C45—C46—C47	137.4 (2)
O1—C11—C12—C17	41.3 (3)	C42—C45—C46—C47	−47.1 (3)
C8—C11—C12—C17	−134.8 (2)	O3—C45—C46—C51	−39.7 (3)
C17—C12—C13—F1	−179.06 (19)	C42—C45—C46—C51	135.8 (2)
C11—C12—C13—F1	−2.1 (3)	C51—C46—C47—F6	177.1 (2)
C17—C12—C13—C14	0.1 (3)	C45—C46—C47—F6	0.0 (3)
C11—C12—C13—C14	177.0 (2)	C51—C46—C47—C48	−1.1 (3)
F1—C13—C14—C15	−179.8 (2)	C45—C46—C47—C48	−178.2 (2)
C12—C13—C14—C15	1.0 (3)	F6—C47—C48—C49	−178.9 (2)
C13—C14—C15—F2	178.9 (2)	C46—C47—C48—C49	−0.6 (3)
C13—C14—C15—C16	−0.6 (4)	C47—C48—C49—F5	−178.9 (2)
F2—C15—C16—C17	179.5 (2)	C47—C48—C49—C50	1.4 (4)
C14—C15—C16—C17	−1.0 (4)	F5—C49—C50—C51	179.8 (2)
C15—C16—C17—C12	2.3 (4)	C48—C49—C50—C51	−0.5 (4)
C13—C12—C17—C16	−1.8 (3)	C47—C46—C51—C50	2.1 (3)
C11—C12—C17—C16	−178.9 (2)	C45—C46—C51—C50	179.4 (2)
C23—C18—C19—C20	2.8 (4)	C49—C50—C51—C46	−1.4 (4)
Cl2—C18—C19—C20	−178.9 (2)	N3—C10—N1—N2	1.5 (3)
C18—C19—C20—C21	−1.1 (4)	N3—C10—N1—C8	177.9 (2)
C19—C20—C21—C22	−2.1 (4)	C7—C8—N1—C10	112.1 (3)
C19—C20—C21—C24	−180.0 (2)	C11—C8—N1—C10	−61.8 (3)
C20—C21—C22—C23	3.7 (4)	C7—C8—N1—N2	−71.9 (3)
C24—C21—C22—C23	−178.4 (2)	C11—C8—N1—N2	114.2 (2)
C21—C22—C23—C18	−2.0 (4)	N3—C9—N2—N1	0.4 (3)
C19—C18—C23—C22	−1.3 (4)	C10—N1—N2—C9	−1.1 (3)
Cl2—C18—C23—C22	−179.6 (2)	C8—N1—N2—C9	−177.9 (2)
C20—C21—C24—C27	−34.9 (4)	N1—C10—N3—C9	−1.2 (3)
C22—C21—C24—C27	147.3 (3)	N2—C9—N3—C10	0.4 (3)
C21—C24—C27—N4	−2.8 (4)	N6—C26—N4—N5	−1.2 (3)
C21—C24—C27—C28	−170.8 (2)	N6—C26—N4—C27	−177.1 (2)
C24—C27—C28—O2	150.8 (2)	C24—C27—N4—C26	−95.4 (3)
N4—C27—C28—O2	−18.0 (3)	C28—C27—N4—C26	73.4 (3)
C24—C27—C28—C29	−25.0 (3)	C24—C27—N4—N5	89.2 (3)
N4—C27—C28—C29	166.23 (18)	C28—C27—N4—N5	−101.9 (2)
O2—C28—C29—C30	−42.7 (3)	N6—C25—N5—N4	−0.8 (3)
C27—C28—C29—C30	133.0 (2)	C26—N4—N5—C25	1.1 (3)
O2—C28—C29—C34	136.6 (2)	C27—N4—N5—C25	177.3 (2)
C27—C28—C29—C34	−47.7 (3)	N4—C26—N6—C25	0.7 (4)
C34—C29—C30—C31	1.9 (3)	N5—C25—N6—C26	0.1 (4)
C28—C29—C30—C31	−178.8 (2)	N9—C44—N7—N8	−0.6 (3)
C29—C30—C31—C32	−1.3 (4)	N9—C44—N7—C42	179.4 (2)
C30—C31—C32—F4	179.5 (2)	C41—C42—N7—C44	−123.7 (3)
C30—C31—C32—C33	−0.4 (4)	C45—C42—N7—C44	50.4 (3)
C31—C32—C33—C34	1.4 (4)	C41—C42—N7—N8	56.2 (3)
F4—C32—C33—C34	−178.5 (2)	C45—C42—N7—N8	−129.6 (2)
C32—C33—C34—F3	−179.4 (2)	N9—C43—N8—N7	0.4 (3)
C32—C33—C34—C29	−0.7 (4)	C44—N7—N8—C43	0.1 (2)
C30—C29—C34—F3	177.8 (2)	C42—N7—N8—C43	−179.89 (19)
C28—C29—C34—F3	−1.5 (3)	N7—C44—N9—C43	0.7 (3)
C30—C29—C34—C33	−0.8 (3)	N8—C43—N9—C44	−0.7 (3)
